# New Medical School

**Published:** 1846-12

**Authors:** 


ARTICLE XVI.

### NEW MEDICAL SCHOOL.

A circular has been issued announcing the organization of the "Medical Department of the University of Buffalo," at Buffalo, N. Y., and that the first annual course of lectures in the institution will commence on the 24th of February next, and continue sixteen weeks.

The Faculty is principally taken from the Geneva school.

We, however, understand that the Geneva school is to be continued under its present organization.

This may be a good arrangement, as students may attend in the school at Geneva during the winter, and at Buffalo in the spring, thus taking two courses of lectures immediately in succession.

The Faculty is organized as follows:—

C. B. Coventry, M. D., *Professor of Physiology and Medical Jurisprudence.*

James Webster, M. D., *Professor of General and Special Anatomy.*

Charles A. Lee, M. D., *Professor of Pathology and Materia Medica.*

James P. White, M. D., *Professor of Obstetrics, and Diseases of Women and Children.*

F. H. Hamilton, M. D., *Professor of Principles and Practice of Surgery and Clinical Surgery.*

Austin Flint, M. D., *Professor of the Principles and Practice of Medicine and Clinical Medicine.*

George Hadley, M. D., *Professor of Chemistry and Pharmacy.*

Corydon La Ford, M. D., *Demonstrator of Anatomy.*

"Fees for all the lectures, is $62, which will, in all cases, be required on taking the ticket."

